# Self-Assembled M_2_L_4_ Nanocapsules: Synthesis, Structure and Host-Guest Recognition Toward Square Planar Metal Complexes ^†^

**DOI:** 10.3390/ma7010287

**Published:** 2014-01-09

**Authors:** Christophe Desmarets, Thierry Ducarre, Marie Noelle Rager, Geoffrey Gontard, Hani Amouri

**Affiliations:** 1 Sorbonne Universités, UPMC Univ Paris 06, Université Pierre et Marie Curie, Institut Parisien de Chimie Moléculaire (IPCM) UMR 8232, 4 place Jussieu, 75252 Paris cedex 05, France; E-Mails: christophe.desmarets@upmc.fr (C.D.); thierry.ducarre@gmail.com (T.D.); geoffrey.gontard@upmc.fr (G.G.); 2 CNRS, Centre National de la Recherche Scientifique, Institut Parisien de Chimie Moléculaire (IPCM) UMR 8232, 4 place Jussieu, 75252 Paris cedex 05, France; 3 NMR Services of Ecole Nationale Supérieure de Chimie de Paris, Institut de Recherche de Chimie Paris (IRCP) UMR 8247, Chimie-ParisTech, 11 rue Pierre et Marie Curie, 75231 Paris Cedex 05, France; E-Mail: marie-noelle.rager@chimie-paristech.fr

**Keywords:** palladium, self-assembly, tetragonal cages, host-guest

## Abstract

Metallosupramolecular cages of the general formulas [M_2_(L)_4_][X]_4_ can be self-assembled in good yields, where M = Pd, X = NO_3_, L = **L^1^** (**1a**); M = Pd, X = OTf, L = **L^1^** (**1b**); M = Pt, X = OTf, L = **L^1^** (**2**); M = Pd, X = OTf, L = **L^2^** (**3**); **L^1^** = 1,3-bis(pyridin-3-ylethynyl)-5-methoxybenzene; and **L^2^** = 2,6-(pyridin-3-ylethynyl)-4-methoxyaniline, respectively. These cages have been fully characterized using ^1^H, ^13^C NMR, elemental analysis, IR spectroscopy, and electrospray mass spectrometry. Additionally the molecular structure of [Pd_2_(**L^1^**)_4_][OTf]_4_ (**1b**) was confirmed using single crystal X-ray diffraction. The capacity of central cavities of M_2_L_4_ cages to accommodate square planar metal complexes was investigated. In particular, the tetracationic cage [Pd_2_(**L^2^**)_4_][OTf]_4_ (**3**) was found to encapsulate the anionic metal complex [PtCl_4_]^2−^ through electrostatic interactions and also via hydrogen bonding with the amino groups of the bridging ligand displayed by this nanocage.

## 1. Introduction

Since the discovery of crown ether, cryptands, and cavitands, prodigious progress in the host-guest chemistry field have been accomplished. For example, during the past few decades, there has been an increasing effort concerning the rational design of new functional metal organic materials, such as discrete coordination cages. These architectures generate tunable shape and size cavities, which offer potential applications as containers for storage, recognition, delivery, or catalysis [1–19]. Recently, it was shown that these hosts are capable of encapsulating inorganic catalysts, larger substrates, and even small peptide sequences [20,21]. Classically, water-soluble polyhedra are effective hosts for aromatic organic guests by hydrophobic effect [12,21–23]. In the same way, negatively charged cages can accommodate cationic organic or complex molecules [24–27]. However, it should be noted that examples reported of positively charged inorganic receptors capable of encapsulating organometallic complexes are limited [28–33]. The molecular recognition property towards the guest relies on the establishment of weak, simultaneous, and selective interactions. Fujita and co-workers reported, for example, metal-metal d-d interaction through the discrete stacking of square planar complexes within a M_4_L_6_ coordination cage [30]. Clever and coworker described the ability of a M_2_L_4_ cage to bind bis(anionic)guest compounds or to form a discrete stack of platinum complexes of the Magnus’ salt type [31–32]. Moreover Crowley and coworkers recently published the preparation of a dipalladium(II) cage complex using 2,6-bis(pyridin-3-ylethynyl)pyridine ligand, capable of binding two molecules of *cis*-platin within its cavity [33]. A strong hydrogen-bonding interaction between the amine ligands of the *cis*-platin guest and a central pyridine moiety of the cage was observed.

In this area, our group has developed the use of semi-rigid bidentate ligand to construct a variety of appealing structures, such as coordination polymers, metallomacrocycles, and cages [34–39]. For example, we have established the synthesis of some metallocryptands and metallocryptates M_2_L_3_, based on Cp*Rh(III) and Cp*Ir(III), as well discrete self-assembled M_2_L_4_ capsules, based on Co(II) and Cu(II), that are capable of encapsulating weakly coordinated anions such as BF_4_^−^ and PF_6_^−^ [34–40]. These anions play a pivotal role as template director to construct such metallocages during the self-assembly process. Molecular recognition takes place via hydrogen bonding—anion or on metal—anion weak interactions and therefore the preparation of these architectures is limited to only weakly coordinating anions. Thus, our efforts were then devoted to prepare related metallocages but with a highly rigid ligand, such as the 1,3-bis(pyridin-3-ylethynyl)-5-methoxybenzene **L^1^**, the latter is more coordinating to the metal ions and recently we have demonstrated that such a ligand leads successfully to the formation of coordination polymers [41]. More recently, we described the synthesis of a novel angular tetradentate rigid ligand, which allowed the preparation of *meso*-helicates with large nonocavities. Pursuing our research in this field, herein, we report the preparation and structural characterization of new tetragonal metal (II) cages of type [M_2_(L)_4_][X]_4_ (**1**–**3**), M = Pd, X = NO_3_, L = **L^1^** (**1a**); M = Pd, X = OTf, L = **L^1^** (**1b**); M = Pt, X = OTf, L = **L^1^** (**2**); M = Pd, X = OTf, L = **L^2^** (**3**) using the rigid assembling bidentate ligands 1,3-bis(pyridin-3-ylethynyl)-5-methoxybenzene (**L^1^**) and 2,6-(pyridin-3-ylethynyl)-4-methoxyaniline (**L^2^**). The solution behavior as well as the capacity of these metallocages to host square planar organometallic complexes are presented and discussed.

## 2. Results and Discussion

### 2.1. Synthesis and Characterization

**Scheme 1.**
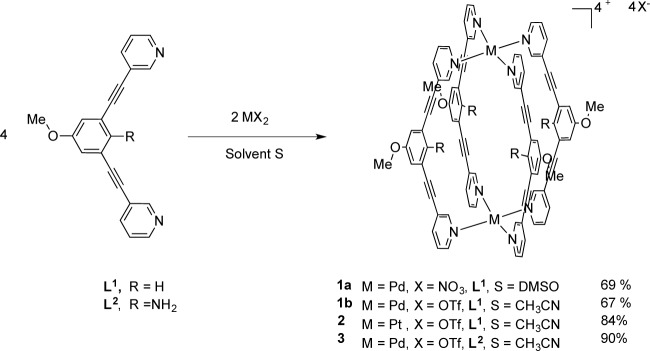
Synthesis of metallosupramolecular cages of type [M_2_(L)_4_][X]_4_.

The bidentate assembling ligands 1,3-bis(pyridin-3-ylethynyl)-5-methoxybenzene **L^1^** and 2,6-(pyridin-3-ylethynyl)-4-methoxyaniline **L^2^** are obtained from our previously described procedure using a Pd/Cu catalyzed Sonogashira cross-coupling reaction [41]. The advantage of these ligands reside in its rigidity and the presence of two ethynylpyridine units to strongly coordinate two metal centers and generate discrete metallocages with cavities instead of polymers assemblies. Treatment of two equivalents of **L^1^** with Pd(NO_3_)_2_·*x*H_2_O in a solution of Dimethyl Sulfoxide (DMSO) at room temperature for 30 min and followed by reaction work-up provided a white microcrystalline precipitate in 69% yield which was characterized as [Pd_2_(**L^1^**)_4_][NO_3_]_4_ (**1a**) (Scheme 1). However, this metallocage is only soluble in DMSO. The solubility properties of such supramolecular architectures being highly dependent on counteranions, we decided therefore to prepare the analogous cage associated to triflate anions. In a similar way, the supramolecular cage [Pd_2_(**L^1^**)_4_][OTf]_4_ (**1b**) was obtained by addition of **L^1^** to an acetonitrile solution of freshly prepared [Pd(CH_3_CN)_4_][OTf]_2_. Moreover, the methodology was extended to platinum which allowed the preparation of metallocage [Pt_2_(**L^1^**)_4_][OTf]_4_ (**2**) in 84% yield. In addition, using the functionalized ligand **L^2^**, the metallocage [Pd_2_(**L^2^**)_4_][OTf]_4_ (**3**) was isolated and characterized as its triflate salt. The later complexes were isolated pure and analyzed elementally and displayed better solubility in some common organic solvent, such as acetonitrile and acetone. The infrared spectrum of these four M_2_L_4_ species showed the presence of the alkyne –C≡C– stretching bands at the range of 2218–2221 cm^−1^ for **1**–**2** and 2205 cm^−1^ for **3**, higher than that observed for the free ligand at 2210 and 2200 cm^−1^ for, respectively, **L^1^** and **L^2^**. Furthermore, a large band attributed to the counter anion (nitrate or triflate) was visible at 1314 cm^−1^ or at the range of 1223–1255 cm^−1^ for the metallocage(s) **1a** and **1b**–**3**, respectively.

**Figure 1.**
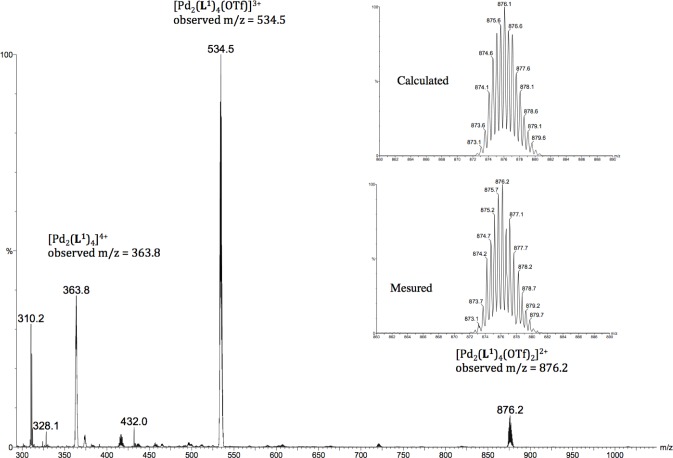
Partial ESI-MS spectrum illustrating the sequence of peaks corresponding to the intact metallocage **1b** associated with different numbers of triflate anions. Experimental and theoretical isotope distribution of [Pd_2_(**L^1^**)_4_ + 2(OTf^−^)]^+^.

**Figure 2.**
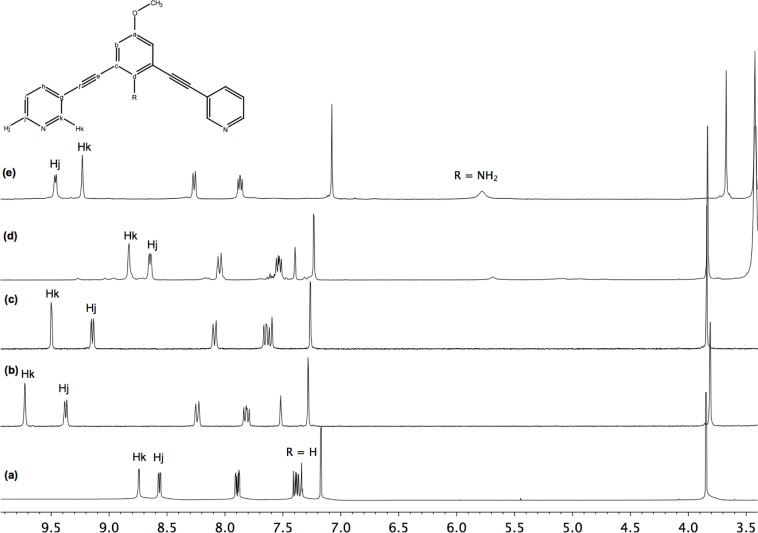
^1^H NMR spectra for (**a**) ligand **L^1^** in CD_3_CN; (**b**) metallocage [Pd_2_(**L^1^**)_4_][NO_3_]_4_ (**1a**) in DMSO-*d*_6_; (**c**) metallocage [Pd_2_(**L^1^**)_4_][OTf]_4_ (**1b**) in CD_3_CN; (**d**) metallocage [Pt_2_(**L^1^**)_4_][OTf]_4_ (**2**) in CD_3_CN and (**e**) metallocage [Pd_2_(**L^2^**)_4_][OTf]_4_ (**3**) in CD_3_CN.

Moreover, these molecular capsules appeared to be stable both in the solid state and in solution the integrity of the cages is maintained. For example, the electrospray mass spectrum of [Pd_2_(**L^1^**)_4_][OTf]_4_ (**1b**) clearly indicates the formation of [PdL_4_] species in association with varying numbers of triflate counterions (Figure 1). The ^1^H NMR spectrum of [M_2_L_4_][X]_4_ cages were recorded either in DMSO-*d*_6_ or CD_3_CN. Upon cage formation with Pd(II) or Pt(II) salts, the spectroscopic data were consistent with the higher symmetry of the assemblies. Furthermore, the formation of the cages induced a downfield shift in the NMR spectrum relative to the free ligand, particularly for H_j_, H_k_ owing to their proximity to the metal center (see Figure 2).

Several attempts have been performed to obtain suitable crystals of **1**–**3** for an X-ray diffraction study. Unfortunately, crystals of sufficient quality could not be obtained for **1a**, **2**, and **3**; however, crystals of cage [Pd_2_(**L^1^**)_4_][OTf]_4_ (**1b**) were grown by vapor diffusion of diethyl ether into a solution of the complex in CH_3_CN (*vide infra*).

#### 2.1.1. X-ray Molecular Structure of [Pd_2_(**L^1^**)_4_][OTf]_4_ (**1b**)

**Figure 3.**
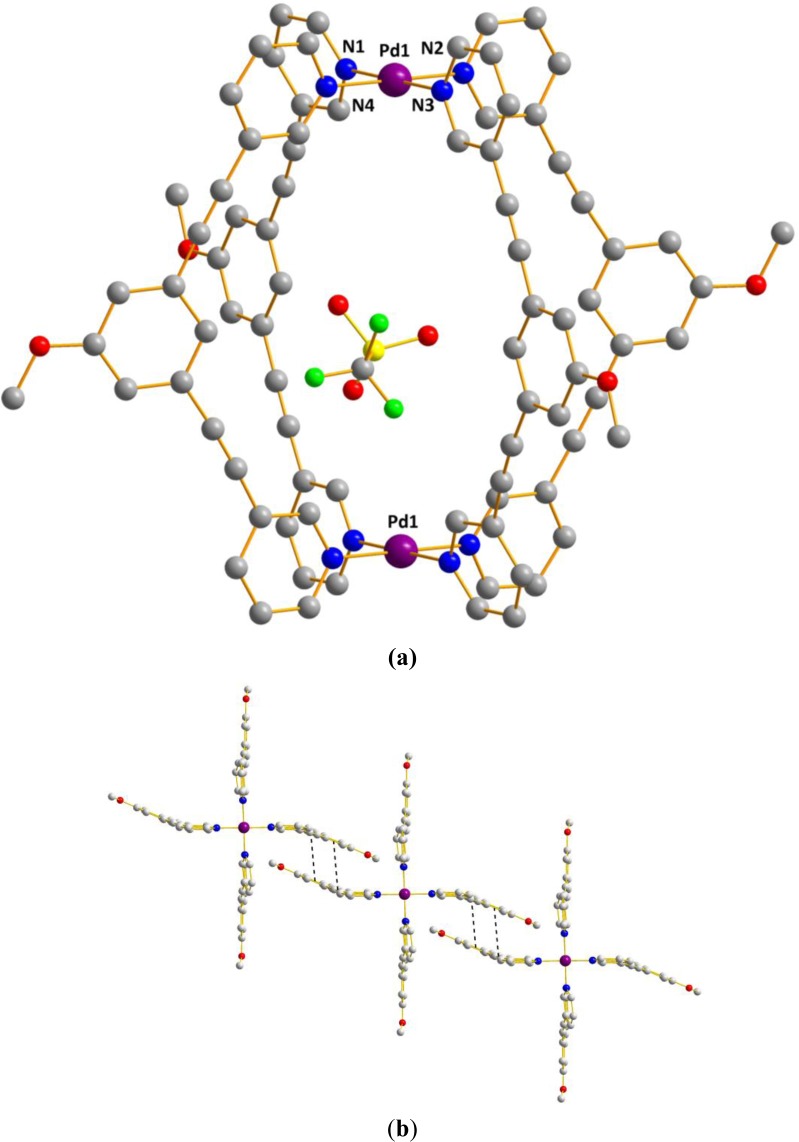
X-ray crystal structure of [Pd_2_(**L^1^**)_4_][OTf]_4_ **1b** (**a**) view of the cationic part of **1b** with one triflate anion within the cavity. (C gray, O red, N blue, Pd violet; H atoms are omitted for clarity). Selected bond lengths (Å) and angles (°): Pd1–Pd1 11.826 (1), Pd–N1 2.025 (4), Pd1–N2 2.024 (4), Pd1–N3 2.025 (4), Pd1–N4 2.023 (4), N1–Pd1–N3 179.01 (15), N2–Pd1–N4 179.65 (15) and (**b**) 1D chain of metallocapsules **1b** generated by π-π contacts among individual units.

The metallocage **1b** crystallized in the triclinic space group *P*-1. The structure shows the formation of a [Pd_2_L_4_] tetragonal cage where each Pd adopts a square planar geometry (Figure 3). The structure shows also that the four rigid bidentate ligands **L^1^** are chelating to two metal centers, thus that each Pd(II) metal center is bound by pyridine of four different bridging ligand **L^1^** (Figure 3a). The Pd–N bond distances lie in the range of 2.023(4) Å to 2.025(4) Å in accord to those reported in the literature for palladium complexes with pyridyl ligands reported by Hooley and others [17,30–33,42]. The Pd–Pd distance is 11.826(1) Å and the average distance between two facing phenyl rings is nearly 11 Å. We also note the presence of π-π interactions (3.333 Å to 3.508 Å) between two individual M_2_L_4_ complexes via the central arene and the acetylene groups of bridging ligand **L^1^** to form a 1D supramolecular chain constituted of 3D capsules. (Figure 3b) Further, solvent molecules and one disordered triflate are present inside the cage cavity, which does not show any interaction with the framework of the nanocage. Then we looked at their solution behavior in order to probe whether the encapsulated triflate anion inside the cavity exchanges with the free ones. Thus a VT ^19^F-NMR study was undertaken.

#### 2.1.2. Variable Temperature ^19^F-NMR Study on [Pd_2_(**L^1^**)_4_][OTf]_4_ (**1b**) and [Pd_2_(**L^2^**)_4_][OTf]_4_ (**3**)

**Figure 4.**
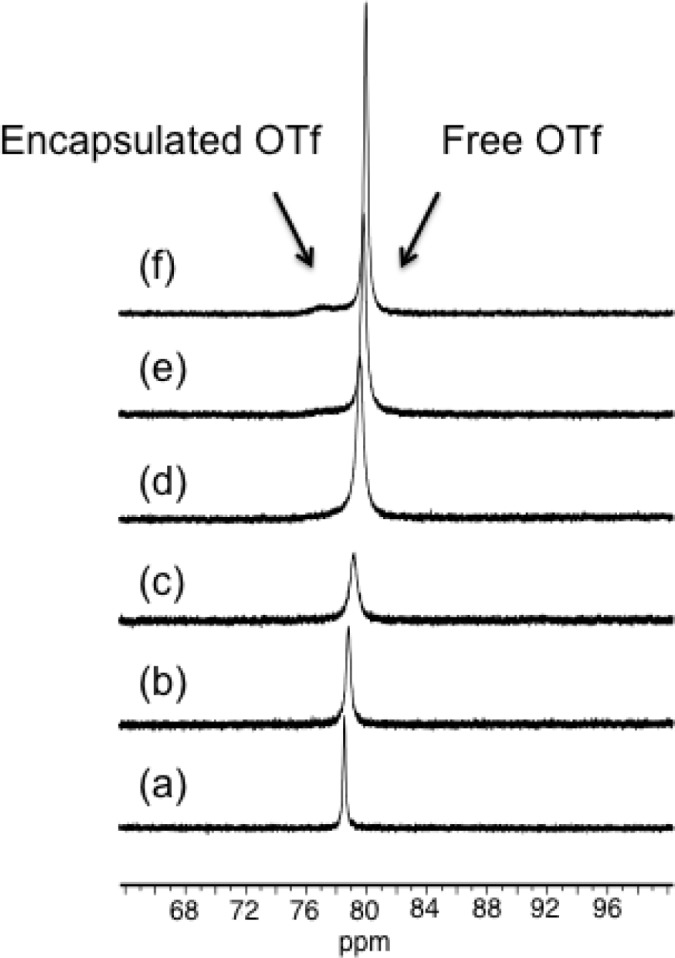
^19^F NMR sprecta of [Pd_2_(**L^2^**)_4_][OTf]_4_ (**3**) cage in CD_3_CN (3.4 mol in 0.5 mL) at variable temperature. (**a**) 25 °C; (**b**) 10 °C; (**c**) 0 °C; (**d**) −10 °C; (**e**) −15 °C and (**f**) −20 °C.

Thus, we first studied the solution behavior of [Pd_2_(**L^1^**)_4_][OTf]_4_ (**1b**) by VT ^19^F NMR in the range from −20 °C to 30 °C. Due to technical limitation we were unable to achieve lower temperature. The ^19^F-NMR spectra recorded in CD_3_CN showed the presence of only one signal at −78.3 ppm suggesting a fast exchange between the encapsulated and the free anions in the range of temperature of study. However, for the metallocage [Pd_2_(**L^2^**)_4_][OTf]_4_ (**3**) displaying amino groups in the assembling ligand **L^2^**, the ^19^F-NMR spectra recorded in CD_3_CN in the above range of temperatures showed that initially the singlet at −78.8 pm broadens upon lowering the temperature and at −20 °C the spectrum displayed a signal at −79.9 ppm and a broad peak centered at −76.4 ppm that we tentatively attribute it to the encapsulated triflate anion (see Figure 4). These data suggest that in metallocage **3**, the encapsulated and free anions are exchanging but at a slower rate compared to that of [Pd_2_(**L^1^**)_4_][OTf]_4_ (**1b**). We feel that, the different behavior of **3** might be attributed to the presence hydrogen bonding interactions between encapsulated triflate and the endohedral amine groups of ligand **L^2^** pointing towards the interior of the cavity, which holds the anion more strongly than in other metallocages with ligand **L^1^** that does not contain any amino groups. These results prompted us to investigate the host-guest properties of these capsules and more particularly towards square planar complexes.

### 2.2. Host-Guest Chemistry

We reasoned that the host-guest recognition is originated from the electrostatic interactions between the anionic guest and the positively charged assembly, furthermore, the presence of amino groups in the bridging ligand should strengthen such interaction, as shown by the ^19^F-NMR study described in the previous section. Due to solubility limitation, the host-guest studies were carried out in DMSO-d_6_ solution.

**Figure 5.**
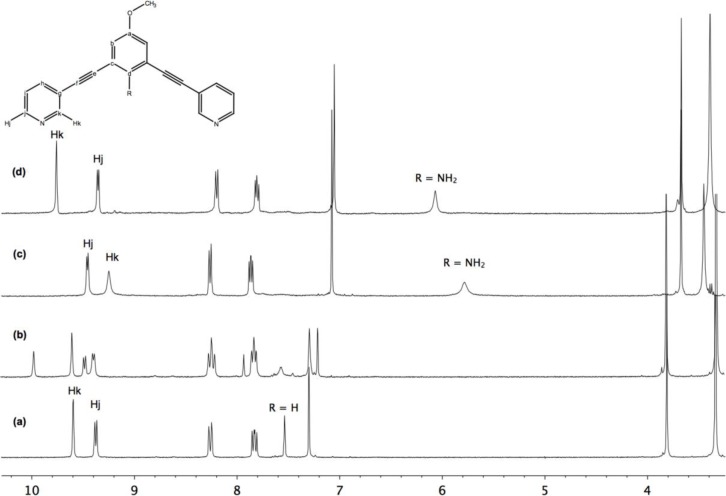
^1^H NMR titration of [PtCl_4_][*n*-Bu_4_N]_2_ inside the metallocages [Pd_2_(**L^1^**)_4_][OTf]_4_ (**1b**) and [Pd_2_(**L^2^**)_4_][OTf]_4_ **3** in DMSO*-d*_6_. (**a**) Cage **1b**; (**b**) addition of one equivalent of guest; **(c)** cage **3** and (**d**) addition of one equivalent of guest.

No changes were observed in the ^1^H NMR spectra upon addition of whether Pd(acac)_2_ or *cis*-platin to a solution of cages **1**–**3**, suggesting that no host-guest binding had occurred with neutral complexes. Despite this unsuccessful results, we investigated other metal complexes but with anionic charge. Thus, when one equivalent of [PtCl_4_][*n*-Bu_4_N]_2_ was added to a DMSO solution of cage [Pd_2_(**L^1^**)_4_][OTf]_4_ (**1b**), the ^1^H NMR spectrum allowed us to identify the formation of a new product relative to the starting material **1b** suggesting partial formation of the host-guest [[PtCl_4_] ⊂ Pd_2_(**L^1^**)_4_][OTf]_2_ complex (Figure 5). Further addition of excess of PtCl_4_^2−^ to the reaction mixture did not achieve full incorporation of one equivalent of the guest as expected, but led to the decomposition of the host-guest supramolecular assembly.

Thus, we then investigated the behavior of the metallocage [Pd_2_(**L^2^**)_4_][OTf]_4_ (**3**), where the assembling ligand **L^2^** contains endohedral amino groups. Addition of one equivalent of [PtCl_4_][*n*-Bu_4_N]_2_ to **3** produced immediate significant peak shifts in the ^1^H-NMR spectrum relative to the starting material, which suggested the exclusive formation of the host-guest complex [[PtCl_4_] ⊂ Pd_2_(**L^2^**)_4_][OTf]_2_ (**4**) (Figure 5). The incorporation of the negatively charged guest inside the cage **3** resulted in a characteristic downfield shift for the inward pointing hydrogen of the amino groups of the metallocage **4**. Furthermore, the signals attributed to hydrogens H_k_, H_j_, on either side of the coordinating nitrogen moved from 9.47 and 9.23 ppm in metallocage [Pd_2_(**L^2^**)_4_][OTf]_4_ (**3**) to 9.37 and 9.77 ppm in [[PtCl_4_]⊂Pd_2_(**L^2^**)_4_][OTf]_2_ (**4**). In addition, as mentioned previously, the downfield shift assigned to the protons of amino groups pointing into the center of the cavity, suggested a strong interaction between NH_2_ and the anionic guest. On the other hand, no changes in the ^1^H NMR spectrum of **4** occurred when leaving the solution to stand over a long period of time, suggesting that this host-guest system is kinetically and thermodynamically stable.

**Figure 6.**
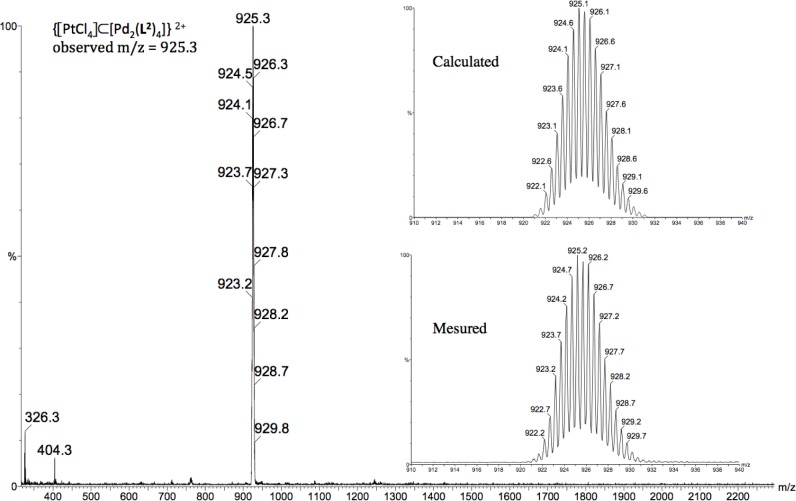
Calculated and observed isotope pattern for the intact metallocage [[PtCl_4_] ⊂ Pd_2_(L2)4][OTf]2 (4).

To confirm the formation of the host-guest system [[PtCl_4_] ⊂ Pd_2_(**L^2^**)_4_][OTf]_2_ (**4**), the reaction was repeated in CH_3_CN, and a pale orange precipitate was then isolated and characterized by ^1^H NMR as [[PtCl_4_] ⊂ Pd_2_(**L^2^**)_4_][OTf]_2_ (**4**). Moreover, the existence of assembly **4** in solution was corroborated by electrospray mass spectroscopy. The +2 charge state of the [PtCl_4_] ⊂ [Pd_2_(**L^2^**)_4_] fragment appears at m/z = 925.3 and was verified by comparison of the observed and theoretical isotopic patterns (Figure 6). Unfortunately, all our attempts to obtain crystals of sufficient quality of [[PtCl_4_] ⊂ Pd_2_(**L^2^**)_4_][OTf]_2_ (**4**) for an X-ray structural determination were unsuccessful.

All over these results are highly important to the area of host-guest chemistry of metallocages as they suggest the necessity to have two complementary effects; namely electrostatic interactions and endohedral hydrogen functionality in the metallocage, which operate in synergy for a successful encapsulation of the kinetically labile metal complex [PtCl_4_]^2−^.

## 3. Experimental Section

### 3.1. General Information and Materials

All solvents used were reagent grade or better. Commercially available reagents were used as received. **L^1^**, **L^2^** and [Pd(CH_3_CN)_4_][OTf]_2_ were prepared according to published methods [41,43]. All experimental manipulations were carried out under argon using Schlenk techniques. IR spectra were recorded on a Bruker Tensor 27 (Bruker Corp., Rheinstetten, Germany) equipped with a Harrick ATR. Elemental analyses were performed by the microanalytical laboratory of Institut de Chimie des Substances Naturelles, Gif-sur Yvette. Positive ESI mass spectra were obtained using a triple quadrupole mass spectrometer (Quattro II Micromass, Waters, UK). Automatic data acquisition was processed using the software *Masslynx V4.0*. NMR experiments were carried out on a Bruker Avance II 300 MHz or Bruker Avance III HD 400 MHz spectrometer operating at 300 K with chemical shifts references to residual solvent peaks. Chemical shifts are reported as parts per million (ppm) and coupling constant (*J*) in hertz (Hz). Standard abbreviations indicating multiplicity were used as follows: m = multiplet; t = triplet; d = doublet; s = singlet and b = broad.

### 3.2. Synthesis

**Synthesis of metallocage [Pd_2_(L^1^)_4_][NO_3_]_4_** (**1a**): Ligand **L^1^** (31.2 mg, 0.1 mmol) was added to a solution of Pd(NO_3_)_2_·*x*H_2_O (87 mg, 0.05 mmol) in DMSO (2 mL). The brown solution was stirred at room temperature 30 min. Hexane and acetone were added (ratio 1:2) to obtain a white-off solid. The solid was collected by filtration and dried under vacuum: 31 mg of white-off solid (69%) characterized as [Pd_4_(**L^1^**)_4_][NO_3_]_4_ **1a**; IR (ATR): (ν, cm^−1^) ν(C≡C)= 2218, ν(N–O)= 1302 cm^−1; 1^H NMR (300 MHz, DMSO-*d*_6_): δ 9.73 (m, 2H, H_k_), 9.37 (d, J = 6 Hz, 2H, H_j_), 8.25 (dd, J = 3, 9 Hz, 2H, H_h_), 7.81 (t, J = 6 Hz, 2H, H_j_), 7.52 (s, 1H, H_d_), 7.28 (s, 2H, H_b_), 3.81 (s, 3H, OMe); ^13^C NMR (75 MHz, DMSO-*d*_6_): δ 162.1, 159.4, 150.1, 153.1, 145.6, 122.9, 122.1, 105.6, 98.7, 93.7, 84.9, 55.7; Anal. calcd for [Pd_2_(**L^1^**)_4_]·[NO_3_]_4_·5DMSO: C 53.94, H 4.14, N 8.03; Found: C 51.39, H 4.04, N 8.14.

**Synthesis of metallocage [Pd_2_(L^1^)_4_]OTf]_4_** **(1b):** Ligand **L^1^** (36.2 mg, 0.116 mmol) was added to a solution of [Pd(CH_3_CN)_4_]OTf]_2_ (33 mg, 0.058 mmol) in CH_3_CN (15 mL). The yellow solution was stirred at room temperature 30 min, during which time the solution initially yellow turns orange. The solvent was removed under vacuum. Hexane (10 mL) was then added. The solids were collected by filtration from the *n*-hexane solution, washed with CH_2_Cl_2_ (5 mL), diethylether (3 × 5 mL), and dried under vacuum: 40 mg of white-off solid (67%) This supramolecular cage was recrystallized from CH_3_CN/Et_2_O to afford quantitatively yellow crystals and was characterized as [OTf⊂(Pd_2_(**L^1^**)_4_][OTf]_3_ **1b**; IR (ATR): (ν, cm^−1^). ν(C≡C) = 2221, ν(OTf) = 1223 cm^−1^, ^1^H NMR (400 MHz, CD_3_CN): δ 9.47 (d, *J* = 1.8 Hz, 2H, H_k_), 9.11 (dd, *J* = 5.9, 1.3 Hz, 2H, H_j_), 8.05 (ddd, *J* = 8.1, 1.8, 1.3 Hz, 2H, H_h_), 7.61 (dd, *J* = 8.1, 5.9 Hz, 2H, H_i_), 7.56 (t, *J* = 1.4 Hz, 1H, H_d_), 7.23 (d, *J* = 1.4 Hz, 2H, H_b_), 3.81 (s, 3H, OMe); ^13^C NMR (100 MHz, CD_3_CN: δ 160.9 (C_a_), 154.1 (C_k_), 151.0 (C_j_), 143.8 (C_h_), 128.4 (C_i_), 127.7 (C_d_), 124.7 (C_g_), 124.2 (C_c_), 119.9 (C_b_), 94.9 (C_e_), 85.1 (C_f_), 56.5 (C_OMe_); ES-MS (m/z): [Pd_2_(**L^1^**)_4_]^4+^: 363.60; found: 363.8, [Pd_2_(**L^1^**)_4_(CF_3_SO_3_)]^3+^: 534.70; found: 534.5, [Pd_2_(**2**)_4_(CF_3_SO_3_)_2_]^2+^: 876.20; found: 875.6; Anal. calcd for [Pd_2_(**L^1^**)_4_.[OTf]_4_.CH_2_Cl_2_: C 50.55, H 3.10, N 5.07; Found: C 50.50, H 3.15, N 5.15.

**Synthesis of metallocage [Pt_2_(L^1^)_4_][OTf]_4_** **(2):** AgOTf (29.1 mg, 0.113 mmol) was added to a acetonitrile solution of [Pt(COD)Cl_2_. The solution was stirred in the dark at room temperature for 10 h, during which time a white precipitate of AgCl formed. The filtrate was transferred to a schlenk via cannula. An acetonitrile solution of ligand **L^1^** (35.4 mg, 0.113 mmol) was then added via cannula to the solution containing [Pt(CH_3_CN)_4_][OTf]_2._ The solution was let to stir at room temperature 3 h, during which time the solution turns pale yellow. The solvent was removed under vacuum. Hexane (10 mL) was then added. The solids were collected by filtration from the *n*-hexane solution, washed with CH_2_Cl_2_ (5 mL), diethylether (3 × 5 mL) and dried under vacuum: 53 mg of pale yellow solid (84%) characterized as [Pt_2_(**L^1^)**_4_][OTf]_4_ **2**; IR (ATR): (ν, cm^−1^) ν(C≡C)= 2220, ν(OTf)= 1255 cm^−1^, ^1^H NMR (300 MHz, DMSO-*d*_6_): δ 8.82 (m, 2H, H_k_), 8.60 (m, 2H, H_j_), 7.96 (m, 2H, H_h_), 7.50 (m, 2H, H_i_), 7.37 (s, 1H, H_d_), 7.18 (s, 2H, H_b_), 3.83 (s, 3H, OMe); ^13^C NMR (75 MHz, DMSO-*d*_6_): δ 154.6, 152.3, 144.0, 135.2, 133.2, 127.5, 122.4, 122.2, 94.2, 85.4, 54.9; Anal. calcd for [Pt_2_(L^1^)_4_][OTf]_4_: C 48.43, H 3.14, N 7.70; Found: C 47.97, H 3.21, N 7.30.

**Synthesis of metallocage [Pd_2_(L^2^)_4_][OTf]_4_** **(3):** Ligand **L^2^** (100 mg, 0.30 mmol) was added to a solution of Pd(CH_3_CN)_4_][OTf]_2_ (87 mg, 0.15 mmol) in CH_3_CN (15 mL). The yellow solution was stirred at room temperature 30 min, during which time the solution initially yellow turns orange. The solvent was removed under vacuum. Hexane (10 mL) was then added. The solids were collected by filtration from the *n*-hexane solution, washed with CH_2_Cl_2_ (5mL), diethylether (3 × 5 mL) and dried under vacuum: 147 mg of orange solid (93%). This supramolecular cage was recrystallized from CH_3_CN/Et_2_O to afford quantitatively yellow crystals and was characterized as [Pd_2_(**L^2^**)_4_][OTf]_4_ **3**; IR (ATR): (ν, cm^−1^) ν(C≡C) = 2205, ν(OTf) = 1236 cm^−1; 1^H NMR (400 MHz, DMSO-*d*_6_): δ 9.46 (d, *J* = 5.8 Hz, 2H, H_j_), 9.23 (s, 2H, H_k_), 8.26 (d, *J* = 8.0 Hz, 2H, H_h_), 7.86 (dd, *J* = 8.0, 5.8 Hz, 2H, H_i_), 7.07 (s, 1H, H_b_), 5.78 (bs, 2H, NH_2_), 3.67 (s, 3H, OMe); ^13^C NMR (100 MHz, DMSO-*d*_6_): δ 152.8 (Ck), 150.2 (Cj), 150.1 (Ca), 145.7 (Cd), 142.6 (Ch), 127.6 (Ci), 123.1 (Cg), 119.9 (Cb), 106.2 (Cc), 92.7 (Ce), 90.2 (Cf), 55.8 (C-OMe); ES-MS (m/z): [Pd_2_(**L^2^**)_4_]^4+^: 78.07; found: 378.5, [Pd_2_(**L^2^**)_4_(CF_3_SO_3_)]^3+^:553.75; found: 554.7, [Pd_2_(**L^2^**)_4_(CF_3_SO_3_)_2_]^2+^: 05.10; found: 906.3, [Pd_2_(**L^2^**)_4_(CF_3_SO_3_)_3_]^+^: 1961.12; found: 1961.10; Anal. calcd for [C_88_H_60_F_12_N_12_O_16_Pd_2_S_4_]·4H_2_O: C 48.43, H 3.14, N 7.70; Found: C 47.97, H 3.21, N 7.30.

**Synthesis of metallocage [[PtCl_4_] ⊂ Pd_2_(L^2^)_4_][OTf]_2_** **(4):** [PtCl_4_][*n*-Bu_4_N]_2_ (57.5 mg, 0.07 mmol) was added to a solution of [Pd_2_(**L^2^**)_4_][OTf]_4_(**3)** (147 mg, 0.07 mmol) in CH_3_CN (5 mL). The solution was stirred at room temperature for 30 min, during which time an orange precipitate formed. The solids were collected by filtration, washed with diethylether (3 × 5 mL), and dried under vacuum: 147 mg of orange solid (93%); This supramolecular cage was characterized as [[Pt(NO_2_)_4_] ⊂ Pd_2_(2)_4_][OTf]_2_ **4**:^1^H NMR (300 MHz, DMSO-*d*_6_): δ 9.77 (m, 2H, H_k_), 9.37 (d, *J* = 6 Hz, 2H, H_j_), 8.22 (d, *J* = 8.1 Hz, 2H, H_h_), 7.83 (dd, *J* = 6, 8.1 Hz, 2H, H_i_), 7.07 (s, 1H, H_b_), 6.08 (bs, 2H, NH_2_), 3.69 (s, 3H, OMe); ES-MS (m/z): [[PtCl_4_] ⊂ Pd_2_(**L^2^**)_4_]^4+^: 925.3; found: 925.1.

### 3.3. NMR Titration Data

For a typical titration experiment, a 6.6 mM solution of cage (3.3 × 10^−3^ mmol) in DMSO-*d*_6_ (0.5 mL) was treated with 3.3×10^−2^ M solution of [PtCl_4_][*n*-Bu_4_N]_2_ (13.6 mg, 1.66 × 10^−2^ mmol, in 0.5 mL) guest, freshly prepared in DMSO-*d*_6_ (0.1 mL), followed by an equilibrium time of 5 min prior to the NMR measurement.

### 3.4. X-Ray Crystal Structure Determination of Metallocage (1b)

X-ray crystal structure determination of metallocage (**1b**): Data was collected on a Bruker Kappa-APEXII. Unit-cell parameters determination, data collection strategy and integration were carried out with the Bruker APEX2 suite of programs. Multi-scan absorption correction was applied [44]. The structure was solved using SIR92 [45] and refined anisotropically by full-matrix least-squares methods using SHELXL-2013 [46]. Crystallographic data (excluding structure factors) for this structure was deposited at the Cambridge Crystallographic Data Centre with the number CCDC 972857. This data can be obtained, free of charge, from the Cambridge Crystallographic Data Centre via www.ccdc.cam.ac.uk.

Crystal data for (**1b**). Yellow crystal, C_84_H_56_N_8_O_4_Pd_2_, 4(CF_3_O_3_S), 5(C_2_H_3_N), 2(CH_4_O), triclinic, *P*-1, a = 12.6470(7) Å, b = 13.1997(7) Å, c = 17.9323(9) Å, α = 94.961(2)°, β = 98.899(2)°, γ = 106.745(2)°, V= 2804.81 Å^3^, Z = 1, T = 200(1) K, μ = 0.483 mm^−1^, 32424 reflections measured, 11364 independent (R_int_ = 0.0326), 8896 observed [I > 2σ(I)], 746 parameters, final R indices R1 [I > 2σ(I)] = 0.0578 and wR2 (all data) = 0.1823, GOF(on F^2^) = 1.076, max/min residual electron density 1.370/−0.817.

## 4. Conclusions

In this paper, we have reported the self-assembly of a family of metallocage of the general formulae [M_2_(L)_4_][X]_4_ (**1**–**3**) in high yield with rigid bidentate ligands **L^1^** and **L^2^**, based on a diethynylpyridine unit. These nanocapsules possess internal cavities and can be utilized to encapsulate guest molecules. We have indeed demonstrated that cage **3** can efficiently bind one square planar metal [PtCl_4_][NBu_4_]_2_ complex within its cavity. In addition to the purely electrostatic interactions between the dicationic cage and the dianionic guest PtCl_4_^2−^, this host-guest interaction is also strengthened by the presence of hydrogen bonding between the amino groups of ligand **L^2^**, which pointed inward of the cavity and the guest. To our knowledge, this is a rare example displaying the encapsulation of a metal-complex within a nanocage. Our results are highly important for the field of host-guest interactions and namely to the study of chemical transformation by metal complexes within confined spaces.

## Supplementary Materials

Supplementary File 1
